# Retrospective Analysis of Burden of Illness of Congenital Pulmonary Valve Disease in a Large, Commercially Insured US Population

**DOI:** 10.36469/001c.156168

**Published:** 2026-03-19

**Authors:** Tariku J. Beyene, Vincent J. Willey, Malvika Venkataraman, Katherine Wong, Eric Anderson, Sophie C. Hofferberth

**Affiliations:** 1 Carelon Research, Wilmington, Delaware; 2 Hopewell Economics, LLC, Springfield, Ohio; 3 Brigham and Women’s Hospital, Harvard Medical School, Boston, MA; 4 Autus Valve Technologies, Inc., Boston, Massachusetts

**Keywords:** congenital pulmonary valve disease, healthcare costs, lifetime medical costs, real-world data

## Abstract

**Background:**

Babies born with congenital pulmonary valve disease (CPVD) face a lifetime burden of disease and significant medical expense beginning in their first year of life.

**Objectives:**

To assess costs and burden of care in patients with CPVD from birth through age 65.

**Methods:**

This retrospective study used administrative claims data to examine healthcare costs and utilization among commercially insured patients with ≥2 claims with CPVD diagnosis between January 1, 2006, and April 30, 2024; the earliest CPVD claim defined the index date. Per-patient-per-year costs were calculated by age category. Mean annual costs for each age group were multiplied by age category duration to estimate full age category period costs. Costs for each age category period were summed to estimate lifetime costs through 65 years of age. Sensitivity analyses were conducted in subgroups with continuous eligibility from birth through 60 months and 120 months.

**Results:**

Among eligible patients (N = 22 751), 53.2% experienced an average of 1.8 CPVD-related inpatient admissions during their first year: mean (standard deviation) cumulative admission days, 50.2 (74.2); with 267 945(737 565;median,6487[interquartilerange(IQR),0−238 486]inassociatedcosts.AmongthosewithinpatientadmissionsforCPVD−relatedproceduresduringchildhood,averageannualcostsrangedfrom97 791 (288 606; 32 029 [15 984-72 762]) to 514 395(926 572;222 373[114 107−530 671]byagecategory.Emergencydepartmentutilizationrangedbetween859 537 (111 982; 13 690 [918-66 625]) and 120 months (N = 174) at 28 893(69 019;2852[400−23 858]).Byage21,projectedcumulativeCPVD−relatedmedicalcostsaveraged788 188 and reached $2.1 million through age 65. Over half of patients had nearly 2 months of hospital stays in their first year, with substantial inpatient costs. The financial burden and demanding care began early and remained steady, reflecting the chronic nature of CPVD.

**Conclusion:**

This study demonstrates a substantial burden of illness and lifetime mosaic of ongoing medical needs for individuals with CPVD and the potential for advances in early life care to reduce this sizable projected lifetime burden.

## INTRODUCTION

Congenital heart disease (CHD), the most common major birth defect, affects more than 40 000 babies born in the United States (US) annually.^1,2^ Currently, an estimated 2.4 million children and adults in the US live with CHD, at an estimated annual healthcare cost of $9.8 billion.^3,4^ Among newborns with CHD, approximately 20% have congenital pulmonary valve disease (CPVD) anomalies that affect the right ventricular outflow tract, including double outlet right ventricle, tetralogy of Fallot (ToF), congenital pulmonary stenosis, and pulmonary atresia.^1,5–7^ Annually, CPVD is diagnosed in over 7000 US births.^8,9^

While some cases involve “watchful waiting,” babies born with CPVD generally require intervention in the first year of life to obviate life-threatening morbidity. Some infants require emergent surgical establishment of the right ventricle to pulmonary artery connection, while the majority undergo palliative surgical repair to relieve pulmonary outflow obstruction.^5,10^ However, this procedure disrupts the native pulmonary valve apparatus and creates severe pulmonary regurgitation.^11^ Consequently, chronic pulmonary insufficiency leads to early-onset, progressive right ventricular enlargement.^12,13^ This volume overload leads to adverse myocardial remodeling, which can result in reduced exercise capacity, poor growth, and other symptoms of right-heart dysfunction during childhood.^12^

To address pulmonary valve dysfunction, patients with CPVD often require eventual pulmonary valve replacement. Unfortunately, existing valve replacement options (eg, off-label bioprosthetic valves, homograft donor valves) used in children often fail early due to multiple factors, including valve oversizing, rapid structural deterioration, thrombosis, infection and outgrowth of the prosthesis.^14–18^ Pediatric patients may face repeated pulmonary valve replacement procedures throughout childhood and adolescence, which carry medical risk and are physically, emotionally, and financially burdensome for patients and families.^6,14,15,17,18^ Therefore, a strategy that minimizes the lifetime number of interventions would be beneficial.^12^

Due to the high risk of early prosthetic valve failure and subsequent need for repeated invasive open-heart operations, clinicians typically only refer children with rapid disease progression for pulmonary valve replacement, while many patients are managed medically and undergo delayed intervention during late adolescence or early adulthood when an adult-sized valve can be implanted.^19^ Delaying treatment significantly increases the risk of irreversible right ventricular dysfunction, leading to reduced quality of life and potential for arrhythmias, heart failure, and premature death in adulthood.^12,20^

Due to cardiovascular morbidity, the burden of caring for patients with CPVD is substantial. While the cost of care has been estimated for recipients of specific CPVD procedures, there is a paucity of published research on the long-term economic impact of CPVD.^21^ This study intends to address this gap in the literature by assessing and estimating lifetime financial costs and burden of care in a real-world population of patients with CPVD from birth through age 65.

## METHODS

### Data Source

This retrospective, observational study used administrative claims data from the Healthcare Integrated Research Database (HIRD). The HIRD contains medical and pharmacy claims from a large, national commercial payor, representing over 90 million members across the US since 2006. Additionally, social drivers of health (SDoH) data collected from the American Community Survey are integrated into the HIRD at the census block group level. Most HIRD members are commercially insured (~90%), with the remainder covered by managed Medicare.^22^ The 2020 HIRD population was representative of the 2020 US census population in terms of sex, age, and geographic region of residence; the race/ethnicity distribution indicates slightly more non-Hispanic White members compared with the census.^23^ Management of all data and study materials conformed with Health Insurance Portability and Accountability Act (HIPAA) rules. A limited dataset, which excluded patient-identifying information, was used under a data use agreement with the covered entities; internal institutional guidelines exempted the research from ethics review based on US Health and Human Services Office for Human Research Protections regulations, including the HIPAA Privacy Rule.

### Study Population

The study population included patients up to 65 years of age with at least 2 medical claims (inpatient, emergency department [ED], or outpatient) with a CPVD diagnosis at least 7 days apart between January 1, 2006, and April 30, 2024. A short (30-day) continuous health plan enrollment criterion was used to minimize potential selection bias associated with length of enrollment. The conditions used to identify CPVD diagnoses using *International Classification of Diseases, Ninth Revision* (ICD-9) or *International Classification of Diseases, Tenth Revision* (ICD-10) diagnostic codes are listed in **Table 1**. The index date was defined as the earliest claim with CPVD diagnosis. As CPVD is a congenital condition, there is no baseline period which could occur prior to the condition. The follow-up period included all claims from index until disenrollment, death, or end of study period (**Supplemental Figure S1**). Subgroups of patients with continuous health plan enrollment from birth through at least 60 months and 120 months, respectively, were included for sensitivity analyses to provide a longer-term analysis for as many individuals as possible.

**Table 1.** Study Attrition

**Step**	**Criteria**	**Patient Counts (%)**
1	Healthcare Integrated Research Database population	93 320 489 (100)
2	Patients with ≥30 days continuous enrollment between 1/1/2006 and 4/30/2024	69 677 013 (74.7)
3	Patients having any code (ICD-9 or ICD-10) for CPVD between 1/1/2006 and 4/30/2024 (% calculated from step 2)	44 132 (0.06)
4	Patients with ≥2 claims of any CPVD diagnosis ≥7 days apart. The earliest claim is the index date (% calculated from step 2): Study population	22 751 (0.03)
	Patients <18 years old at index (from step 4)	14 609 (64.2)
	Patients ≥18 years and older at index (from step 4)	8142 (35.8)
5a	Patients with ≥60 months of follow-up (index to death, disenrollment, or study period) with an age at index date of <1 year old from step 2 and had eligibility at birth	872 (3.83)
5b	Patients with ≥120 months of follow-up (index to death, disenrollment, or study period) with an age at index date of <1 year old from step 2 and had eligibility at birth	174 (0.76)
6	By ICD-9 or ICD-10 diagnosis code (not mutually exclusive) from step 4	
	Patients with ≥2 claims for double outlet right ventricle (ICD code: 745.11 or Q20.1)	2212 (9.7)
	Patients with ≥2 claims for tetralogy of Fallot (ICD code: 745.2 or Q21.3)	8406 (36.9)
	Patients with ≥2 claims for pulmonary valve atresia (ICD code: 746.01 or Q22.0)	2024 (8.9)
	Patients with ≥2 claims for congenital pulmonary valve stenosis (ICD code: 746.02 or Q22.1)	10 535 (46.3)
	Patients with ≥2 claims for other congenital malformations of pulmonary valve (ICD code: 746.00 or Q22.3)	1209 (5.3)
	Patients with ≥2 claims for pulmonary infundibular stenosis (ICD code: 746.02 or Q24.3)	5873 (25.8)
	Patients with ≥2 claims for atresia of pulmonary artery (ICD code: 747.31 or Q25.5)	2318 (10.2)

Abbreviations: CPVD, congenital pulmonary valve disease; ICD-9, *International Classification of Diseases, Ninth Revision*; ICD-10, *International Classification of Diseases, Tenth Revision*.

### Outcome Measures

Demographics including sex, race/ethnicity, geographic region, SDoH, and selected comorbid conditions of interest were captured. SDoH were reported as the socioeconomic status (SES) index from the ACS.

Healthcare resource utilization (HCRU) and healthcare costs, calculated from medical and pharmacy claims, were reported by categories: inpatient, ED, outpatient, and prescription medications. Healthcare costs, calculated from the total amount allowed for paid claims, represent both health plan and patients’ payments. HCRU and costs were reported as all-cause and CPVD-related. All-cause HCRU and costs included all claims regardless of diagnosis, procedure performed, or medication dispensed or administered. CPVD-related HCRU and costs included claims with the CPVD diagnoses used to define the cohort and related diagnoses (**Supplemental Table S1**).^12,24–29^ Additionally, specific CPVD-related procedures were included in CPVD-related HCRU/costs regardless of the diagnosis code associated with those procedure claims (**Supplemental Table S1**).

The age when individuals with CPVD are identified and followed in claims depends on when they are diagnosed and their health plan enrollment period; hence, we captured CPVD patients at varying ages and time horizons. To estimate lifetime costs associated with CPVD, age-based subgroups were adapted from published studies as follows: infants (<1 year), toddlers (1-4 years), children (5-12 years), adolescents (13-21 years), young adults (22-44 years), and middle-aged/older adults (45-65 years).^4,14,30,31^

### Statistical Analysis

Demographics, baseline clinical characteristics, and outcomes of interest were described in univariate statistics. Categorical variables were described using frequency and percentage. Continuous variables were described with measures of centrality (means) and variance (SD), as well as medians and interquartile ranges (IQR).

All-cause and CPVD-related HCRU and costs were calculated per-patient-per-year by age category. Annual costs for each age group were multiplied by number of years within that category to estimate costs within the full age category time period. The costs for each full age category time period were summed to estimate lifetime costs through age 65 years. For the 60-month and 120-month sensitivity analyses, costs were captured for the entire duration of follow-up. Costs were adjusted to 2023 dollars based on the Consumer Price Index of the US Bureau of Labor Statistics for medical care. Sample selection, creation of analytic variables, and analyses were performed using the Instant Health Data platform (Panalgo).

## RESULTS

### Study Population

All eligibility criteria were met by 22 751 individuals with CPVD; overall, the prevalence of CPVD was 0.03% (22 751/69 677 013) (**Table 1**). The most common CPVD diagnoses included congenital pulmonary valve stenosis (N = 10 535; 46.3%) and ToF (N = 8406; 36.9%). The study population had a mean (SD; median [IQR]) duration of follow-up of 3.5 years (3.3; 2.5 [1.2-4.9]) (**Table 2**). For the sensitivity analyses, among individuals who met all eligibility criteria, 872 individuals had at least 60 months of continuous follow-up from birth and 174 individuals had at least 120 months of continuous follow-up from birth. The 60-month subgroup had a mean duration of follow-up of 8.1 years (2.80; 7.3 [6.0-9.4]) and the 120-month subgroup had a mean duration of follow-up of 12.7 years (2.44; 11.9 [10.6-14.2]) (**Supplemental Table S2**).

**Table 2.** Baseline Demographic, Social Drivers of Health, and Clinical Characteristics

**Characteristic**	**Overall**
No. of patients	22 751
Length of follow-up (y), mean (SD); median [IQR]	3.5 (3.3); 2.5 [1.2-4.9]
Age categories (y), n (%)	
<1	5952 (26.2)
1-4	2813 (12.4)
5-12	3825 (16.8)
13-21	3181 (14.0)
22-44	4771 (21.0)
45-65	2209 (9.7)
Gender, n (%)	
Female	11 718 (51.5)
Male	11 030 (48.5)
Geographic region, n (%)	
West	5289 (23.2)
South	7 117 (31.3)
Northeast	3447 (15.2)
Midwest	5539 (24.3)
Unknown	1359 (6.0)
Individual-level race/ethnicity, those with data, n (%)	16 968 (74.6)
American Indian or Alaska Native^a,b^	30 (0.2)
Asian^a,b^	1052 (6.2)
Black or African American^a,b^	1176 (6.9)
Hispanic or Latino of any race^a^	1370 (8.1)
Native Hawaiian or other Pacific Islander^a,b^	21 (0.1)
White^a,b^	12 787 (75.4)
Other race^a,b^	532 (3.1)
Unknown or undisclosed	5783 (25.4)
Area-level SES index^c^ category, n (%)	
1	2454 (10.8)
2	4203 (18.5)
3	5702 (25.1)
4	8009 (35.2)
Missing/unknown	2383 (10.5)
Selected comorbidities,^d^ n (%)	
Hypertension	3812 (16.8)
Heart failure	3505 (15.4)
Developmental delays	3489 (15.3)
Asthma	3382 (14.9)
Dyslipidemia	2746 (12.1)
Ventricular arrythmia	2458 (10.8)
Obesity	2134 (9.4)
Pulmonary hypertension	1941 (8.5)
Ischemic heart disease	1718 (7.6)
Atrial fibrillation	1621 (7.1)
Atrial tachycardia	1540 (6.8)
Chronic heart failure	1270 (5.6)
Cyanosis	1266 (5.6)
Diabetes mellitus	1047 (4.6)
Renal disease	872 (3.8)
Stroke	801 (3.5)
Chronic obstructive pulmonary disease	770 (3.4)
Peripheral vascular disorders	711 (3.1)
Myocardial infarction	438 (1.9)
Sudden cardiac death	402 (1.8)
Liver congestion/fibrosis	391 (1.7)
Right heart failure	332 (1.5)
Arrhythmias (unspecified)	213 (0.9)

Abbreviations: IQR, interquartile range; SD, standard deviation; SDoH, social drivers of health; SES, socioeconomic status.

^a^Percentage among those with data.

^b^Not Hispanic or Latino.

^c^SES index is a composite measure based on 7 SDoH variables. A score of 4 indicates top 25% of SES and a score of 1 indicates bottom 25% of SES using all census block groups and 2017 as reference basis. Patients unable to be linked to SDoH data or with ≥1 of the 7 variables missing are categorized as unknown.

^d^Identified by ≥1 diagnosis code for the condition.

### Population Demographic Characteristics

The study population was balanced between males (48.5%) and females (51.5%) (**Table 2**). The largest age categories were <1 year of age (26.2%) and 22-44 years (21.0%). Geographically, the population was concentrated in the South (31.3%). Race and ethnicity, available for approximately 75% of the population, included White (75.4%), Hispanic or Latino of any race (8.1%), Black or African American (6.9%), and Asian (6.2%). SES data were available for 89.5% of the study population. Most patients (>60%) were determined to be in the top 2 SES index quartiles (**Table 2**). Demographic characteristics for the 60-month and 120-month subgroups are displayed in **Supplemental Table S2**.

### Population Clinical Characteristics

Nearly half of patients presented with congenital pulmonary valve stenosis, with ToF ranking second (**Table 1**). The 3 most common comorbidities were hypertension (16.8%), heart failure (15.4%), and asthma (14.9%) (**Table 2**). Further details about the prevalence of the CPVD diagnoses for each of the subgroups are shown in **Supplemental Table S3**. Arrhythmias, including ventricular arrhythmia (10.8%), atrial fibrillation (7.1%), and atrial tachycardia (6.8%) were prevalent (**Table 2**). Developmental delays were also common (15.3%). Among the 60-month and 120-month subgroups, the most common comorbidities were developmental delays (39.6% and 33.3%), asthma (26.6% and 31.6%), and heart failure (21.9% and 21.3%, respectively) (**Supplemental Table S2**).

### Economic and Utilization Findings

**Figure 1.**
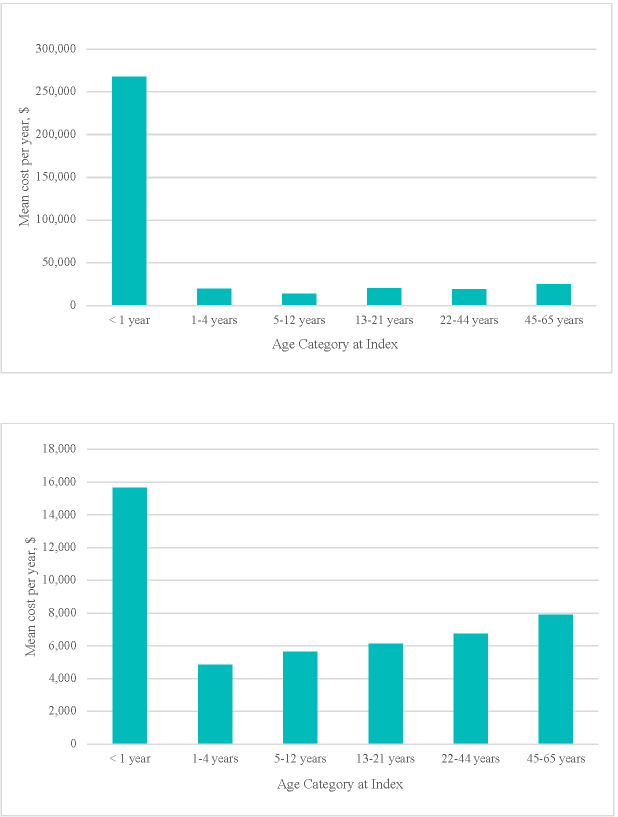
**(a)** CPVD-Related Mean Annual Inpatient Costs by Age at Index; **(b)** CPVD-Related Mean Annual Outpatient Costs by Age at Index Abbreviation: CPVD, congenital pulmonary valve disease.

**CPVD-related:** CPVD-related inpatient admissions were present among 53.2% of patients under 1 year of age, with a mean (SD); median [IQR]) of 1.8 (5.89; 1.0 [0.0-2.0]) visits among all patients and 50.2 (74.21; 22.4 [10.0-60.0]) cumulative admission days among those with inpatient utilization. Heavy utilization (14.6%-34.0%) and lengthy admissions (>6 days) were observed among all ages (**Table 3**). Inpatient utilization frequency by diagnostic group was highest among patients with double outlet right ventricle, ToF, and pulmonary atresia, experienced by about 90% of the <1 year subgroup and over 97% of patients with those diagnoses in the 60-month follow-up subgroup (**Supplemental Table S3**). Inpatient utilization contributed the greatest proportion of total mean (SD; median [IQR]) annual costs for all age categories, ranging from $13 564 (127 445; 0 [0-0]) to $267 954 (737 565; 6,487 [0-238 486]) (**Figure 1a, Table 3**). Surgical interventions were conducted for 19.3% of CPVD patients <1 year, and 6.3% to 10.5% of all other age categories (**Table 4**). These patients incurred substantial mean inpatient costs (range $94 788 (181 583; 38 872 [17 183-81 097]) to $514 385 (926 572; 222 373 [114 107-530 671])) per hospitalization associated with lengthy hospital stays (range 5.7 (9.1; 5.0 [2.0-6.0]) to 24.6 (39.5; 10.0 [6.0-24.0]) days) (**Table 4**).

**Table 3.** Annual Utilization and Costs for CPVD-Related Medical Care by Age Category at Index

**Cost/Utilization**	**Age Category (Years)**					
**<1 (N = 5952)**	**1-4 (N = 7111)**	**5-12 (N = 6560)**	**13-21 (N = 4985)**	**22-44 (N = 5901)**	**45-65 (N = 2298)**	
**Inpatient^a^**						
Patients with ≥1 inpatient admission, N (%)	3169 (53.2)	1364 (19.2)	957 (14.6)	1000 (20.1)	1536 (26.0)	781 (34.0)
Admissions/year						
Mean (SD)	1.8 (5.89)	0.2 (0.67)	0.1 (0.89)	0.2 (2.65)	0.3 (1.36)	0.4 (1.62)
Median [IQR]	1.0 [0.0-2.0]	0.0 [0.0-0.0]	0.0 [0.0-0.0]	0.0 [0.0-0.0]	0.0 [0.0-0.1]	0.0 [0.0-0.3]
Admission days, cumulative						
Mean (SD)	50.2 (74.21)	8.4 (21.11)	7.1 (25.47)	6.3 (32.08)	8.2 (21.30)	9.6 (24.30)
Median [IQR]	22.4 [10.0-60.0]	2.8 [1.3-6.3]	2.0 [0.9-5.0]	1.7 [0.8-4.3]	2.4 [1.1-6.2]	2.6 [1.0-7.0]
Total cost,^b^ $						
Mean (SD)	267 945 (737 565)	19 831 (175 272)	13 564 (127 445)	20 208 (365 434)	19 090 (113 598)	25 106 (117 857)
Median [IQR]	6487 [0-238 486]	0 [0-0]	0 [0-0]	0 [0-0]	0 [0-1 345]	0 [0-7678]
**ED**						
Patients with ≥1 ED visit, N (%)	506 (8.5)	692 (9.7)	533 (8.1)	612 (12.3)	886 (15.0)	481 (20.9)
Total cost,^b^ $						
Mean (SD)	528 (4717)	180 (1349)	189 (2107)	380 (4389)	517 (4222)	700 (4011)
Median [IQR]	0 [0-0]	0 [0-0]	0 [0-0]	0 [0-0]	0 [0-0]	0 [0-0]
**Outpatient^c^**						
Patients with ≥1 outpatient service, N (%)	5866 (98.6)	6361 (89.5)	5855 (89.3)	4692 (94.1)	5620 (95.2)	2231 (97.1)
Total cost,^b^ $						
Mean (SD)	15 637 (33 652)	4833 (13 039)	5639 (20 367)	6135 (27 025)	6747 (19 436)	7911 (26 126)
Median [IQR]	5724 [2251-15 841]	1057 [317-3 379]	1474 [456-4 329]	1720 [573-4 686]	2129 [705-5 486]	2138 [634-6 486]
**Pharmacy**						
Patients with ≥1 pharmacy prescription fill, N (%)	296 (5.0)	262 (3.7)	233 (3.6)	229 (4.6)	368 (6.2)	321 (14.0)
Total cost,^c^ $						
Mean (SD)	165 (2595)	253 (3754)	452 (7813)	465 (7547)	543 (9195)	1093 (11 073)
Median [IQR]	0 [0-0]	0 [0-0]	0 [0-0]	0 [0-0]	0 [0-0]	0 [0-0]
Total costs^b^						
Total annual cost,^b^ $						
Mean (SD)	284 276 (740 975)	25 117 (178 350)	19 844 (130 927)	27 188 (370 163)	26 898 (117 892)	34 833 (124 953)
Median [IQR]	34 541 [3800-⁠263 674]	1283 [364-⁠6 819]	1758 [516-⁠6 813]	2292 [701-⁠9 874]	3472 [1008-⁠14 529]	4075 [878-⁠22 326]
**Years in cohort**	**1**	**4**	**8**	**9**	**23**	**21**
**Projected costs per age category, $**	**284 276**	**100 468**	**158 752**	**244 692**	**618 654**	**731 493**
**Projected lifetime^d^ CPVD-related healthcare costs^a^ through age 65: $2 138 335**						

Abbreviation: CPVD, congenital pulmonary valve disease; ED, emergency department; IQR, interquartile range; SD, standard deviation.

^a^Admissions per year are counted among the total number of members in the respective cohorts; cumulative admission days are counted among patients with at least 1 inpatient admission.

^b^Total costs are calculated per year for all members in the respective cohorts.

^c^Outpatient includes office visits, procedures, texts, medication-related services, durable medical equipment, and various therapy services. Outpatient services are detailed in **Supplemental Table S4**. All costs are adjusted to 2023 US dollars provided by the Bureau of Labor Statistics.

^d^Projected lifetime costs = (Years in each age cohort) × (Total mean cost/Year) and summed for all time periods.

**Table 4.** CPVD Procedure–Related Inpatient HCRU and Costs by Index Age Category

	**Age Category (Years)**					
**<1 (N = 5952)**	**1-4 (N = 7111)**	**5-12 (N = 6560)**	**13-21 (N = 4985)**	**22-44 (N = 5901)**	**45-65 (N = 2298)**	
CPVD procedure^a^–related inpatient utilization						
Patients with ≥1 visit, n (%)	1148 (19.3)	445 (6.3)	432 (6.6)	490 (9.8)	547 (9.3)	242 (10.5)
Length of stay days per admission^b^						
Mean (SD)	24.6 (39.5)	13.5 (35.8)	6.4(8.9)	5.7(9.1)	7.7(12.2)	7.2(8.0)
Median [IQR]	10.0 [6.0-24.0]	6.0 [4.0-10.0]	5.0 [3.0-6.0]	5.0 [2.0-6.0]	5.0 [3.0-7.5]	6.0 [3.0-8.0]
CPVD procedure–related inpatient costs^c^ among those who had the procedure						
CPVD procedure–related inpatient costs, $						
Mean (SD)	514 385 (926 572)	151 187 (616 818)	97 791 (288 606)	123 745 (1 126 640)	99 951 (284 638)	94 788 (181 583)
Median [IQR]	222 373 [114 107-⁠530 671]	42 166 [22 174-⁠101 370]	32 029 [15 984-⁠72 762]	28 583 [13 981-⁠66 347]	36 813 [17 082-⁠89 708]	38 872 [17 183-⁠81 097]
Surgical pulmonary valve replacement–related inpatient utilization						
Length of stay days per admission^b^						
Mean (SD)	20.4 (24.20)	9.2 (11.70)	6.1 (5.08)	6.0 (5.74)	8.2 (10.20)	8.9 (8.68)
Median [IQR]	9.5 [6.4-21.5]	6.0 [5.0-8.0]	5.0 [4.0-6.0]	5.0 [4.0-6.0]	6.0 [5.0-8.0]	7.0 [5.0-9.0]
Surgical pulmonary valve replacement–related inpatient costs among those who had the procedure						
Surgical pulmonary valve replacement–related inpatient costs, $						
Mean (SD)	271 317 (332 187)	199 256 (291 332)	133 201 (154 174)	131 296 (93 331)	154 476 (186 337)	163 884 (102 309)
Median [IQR]	154 508 [111 457-⁠295 048]	122 986 [82 969-⁠183 764]	104 825 [67 723-⁠151 236]	113 973 [81 272-⁠162 168]	114 680 [86 150-⁠162 921]	141 140 [95 494-⁠205 651]

Abbreviations: CPVD, congenital pulmonary valve disease; IQR, interquartile range.

^a^Specific procedures of interest for CPVD procedure–related include the following: (1) primary surgical repair of anatomic defect; (2) surgical/transcatheter pulmonary valve replacement; (3) surgical implantation of right ventricle to pulmonary artery conduit; (4) surgical patch plasty of left or right pulmonary artery; (5) percutaneous balloon pulmonary valvuloplasty; (6) percutaneous branch pulmonary artery balloon angioplasty; (7) percutaneous branch pulmonary artery stent or right ventricular outflow tract stent placement; (8) surgical repair of pulmonary valve; (9) pulmonary valvuloplasty; (10) intraoperative pulmonary valve balloon dilation; (11) main pulmonary artery patch plasty; (12) right ventricular outflow tract reconstruction/repair.

^b^Length of stay days per admission is calculated among those with inpatient admission.

^c^Costs are adjusted to 2023 US dollars from the most recent medical care price index information provided by the Bureau of Labor Statistics.

Outpatient service utilization, nearly universal during the first year of life (98.6%), remained high, averaging nearly 90% or higher for each age cohort (**Table 3**). **Supplemental Table S4** provides a more detailed breakdown of outpatient components. Notably, patients <1 year had a mean (SD; median [IQR]) of 18.7 (32.1; 9.3 [4.0-22.5]) outpatient visits, including 8.1 (13.2; 4.9 [2.3-10.1]) office visits and 6.4 (15.3; 4.0 [2.0-7.8]) imaging encounters during their first year, while outpatient visits in the other age categories ranged between 5.4 (19.8; 2.7 [1.2-5.4]) and 9.6 (15.7; 4.8 [2.1-10.9]) (**Supplemental Table S4**). Outpatient costs, following the pattern of utilization, contributed a heavy annual financial burden for all age categories [mean (SD) range $4833 (13 039) to $15 637 (33 652)] (**Figure 1b, Table 3**). SD values were considerably higher than mean values for all cost and age categories indicating expensive outlier(s) within the data.

### Sensitivity Analysis

CPVD-related inpatient utilization was common among the 60-month subgroup (60.6%) and 120-month subgroup (52.3%), with associated observed mean (SD; median [IQR]) costs of $52 993 (104 694; 9780 [0-55 945]) and $25 077 (63 096; 313 [0-19 019]), respectively. Outpatient service utilization was nearly universal among the 60-month subgroup (99.8%) and 120-month subgroup (99.4%). Associated annual outpatient costs were $6028 (11 917; 1742 [626-6482]) and $3301 (7923; 711 [302-2663]), respectively (Supplemental Table 5). Total observed cost per year was $59 537 (111 982; 13 690 [918-66 625]) and $28 893 (69 019; 2852 [400-23 858]), respectively.

All-cause utilization was prevalent among the 60-month and 120-month subgroups. All-cause mean (SD; median [IQR]) observed total costs were $72 846 (128 710; 20 085 [4,254-81 413]) for the 60-month subgroup and $41 390 (86 262; 8185 [2402-36 376]) for the 120-month subgroup (**Supplemental Table S6**).

### Estimated Lifetime Cost Burden Projection

**Figure 2.**
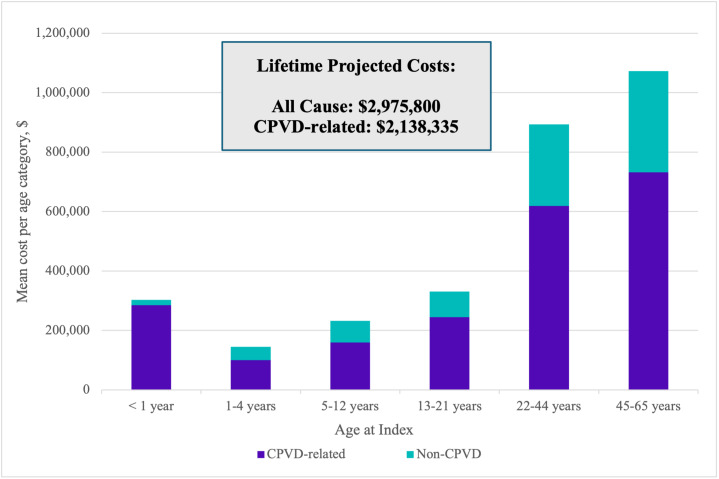
Projected All-Cause Total Costs Shown as Proportions of CPVD-Related and Non-CPVD-Related Costs by Age at Index Abbreviation: CPVD, congenital pulmonary valve disease.

**CPVD-related:** Total CPVD-related mean (SD; median [IQR]) observed annual costs ranged from $19 844 (130 927; 1758 [516-6813]) to $284 276 (740 975; 34 541 [3800-263 674]), depending on age category (**Table 3**). Extrapolating the data, through age 21, the total mean CPVD-related projected cost was $788 188 and through age 65, $2 138 335 (**Figure 2**).

**All-cause:** All-cause mean (SD; median [IQR]) observed annual costs ranged from $29 002 (138 781; 5028 [1995-6538]) to $303 043 (747 650; 53 743 [9619-289 930]), depending on age category (**Supplemental Table S7**). Extrapolated all-cause projected costs across age cohorts totaled $2 975 800 through age 65. All-cause costs were mainly driven by CPVD-related expenses which accounted for 94% of total costs for the first year of life, and approximately 70% of total costs in other age categories (**Figure 2**).

## DISCUSSION

Within a large, commercially insured population, this study demonstrates a substantial overall burden of illness and estimated financial cost for patients with CPVD. Patients and their families face a lifetime of CPVD-related challenges. Previously published estimates of CHD prevalence are derived from birth prevalence and survival statistics; the present study provides real-world evidence of prevalence, long-term costs, and burden of care for patients living with CPVD.^3^

CPVD patients and their families face numerous doctor visits, inpatient stays, outpatient hospital visits, and ED visits, which deleteriously impact quality of life by interrupting family, school, and occupational activities. Additionally, the burden of disease components of ongoing pain, discomfort, reduced exercise capacity, and fatigue warrant consideration.^12^ The childhood and projected lifetime financial burden noted in this study was substantial. Annual all-cause costs among adults in the study were $38 818 for ages 22 to 44 years and $51 055 for ages 45 to 65 years, with about 30% attributed to non-CPVD claims. For context, these values outpace the general annual all-cause healthcare expenditure of the adult US population aged 19 to 64, which ranged between $8313 and $9989 in 2020.^30^ It was beyond the scope of this study to determine the root cause of the high non-CPVD cost for this population. While non-CPVD costs play a role in overall projected lifetime healthcare costs, CPVD-related expense remains a major cost driver.

To address the gap in the present body of published literature about the long-term economic impact of CPVD, observed data in this rare condition were used to extrapolate lifetime costs. While this method may bias annualized cost estimates due to truncated observation windows, the average length of follow-up for study members was 3.5 years, providing the recommended medium to long-term interval of longitudinal data for such extrapolations.^32^ Additionally, the sensitivity analysis subgroups displayed a complete view of 60 months and 120 months of continuous observation from birth. The mean total annual all-cause costs for these subgroups were observed in the same range as the data collected for the age at index categories, supporting the plausibility of the projected lifetime cost result.

Almost half of the study population had congenital pulmonary stenosis and were likely to be managed with watchful waiting, while nearly 10% were diagnosed with pulmonary atresia, which typically requires immediate palliation and future reinterventions.^13^ ToF was highly prevalent within the study population and generally requires surgical intervention within the first few months of life. Improvements in surgical management of ToF have allowed over 98% of patients who survive their first year to survive into adulthood.^11,33^ However, post-CPVD repair pathologies, well established in the literature, frequently impact long-term health; hence, current efforts to advance treatment approaches are directed at preserving lifetime right-ventricular function.^11,13,19,33–35^ By finding substantial burden of illness, both in utilization and expenses, this study demonstrates that standard CPVD interventions continue to have significant limitations which are evident over a patient’s lifetime. Because CPVD patients incur an ongoing cost burden, decision makers should consider the impact of early interventions on long-term health outcomes and costs.

This study was subject to limitations. Administrative data, collected for billing and reimbursement purposes, may be subject to coding errors and omissions. For example, diagnosis codes may be included as rule-out criterion; however, at least 2 CPVD codes were required to reduce false positives. Providers may not prioritize coding an encounter for a congenital defect when treating a patient for conditions which are common sequelae of CPVD, especially in older individuals. In an attempt to capture appropriate CPVD-related utilization and costs, some of these conditions have been included as CPVD-related in the analysis. It is possible that these costs could be attributed to other non-CPVD cardiovascular comorbidities. Lifetime costs were estimated based on costs associated with each age band as projections and not as observed cost because no patient was tracked throughout their lifetime. Study results may not be generalizable to certain population segments: commercially insured individuals may have different characteristics than those who are uninsured or with other types of health insurance such as Medicaid.

## CONCLUSION

Although significant research has improved our understanding of ongoing medical care required for patients with congenital pulmonary valve anomalies, this study retrospectively reveals the substantial financial burden and the treatment burden experienced by individuals with CPVD from infancy through adulthood. Surgical management of CPVD has improved, allowing survival into adulthood for most patients who survive their first year. However, substantial cardiovascular morbidity persists in many CPVD patients. Ongoing CPVD-related utilization and associated expenses demonstrate the need and potential for advances in care in this population.

### Disclosures

T.J.B., V.J.W., M.V., and K.W. are employees of Carelon Research, which received research funding from Autus Valve Technologies, Inc. for the study. T.J.B., V.J.W., and K.W. are stockholders of Elevance Health, the parent company of Carelon Research. E.A. reports consulting services to Autus Valve Technologies, Inc. S.C.H. is an employee, intellectual property holder, stockholder, and co-founder of Autus Valve Technologies, Inc.

## Supplementary Material

Online Supplementary Material
