# Supplementary material for: Retrospective Analysis of Burden of Illness of Congenital Pulmonary Valve Disease in a Large, Commercially Insured US Population
Source: J Health Econ Outcomes Res. 2026 Mar 19;13(1):56–65. doi: 10.36469/001c.156168 (PMC13005609; doi:10.36469/001c.156168)
Supplement: Online Supplementary Material [file jheor_2026_13_1_156168_335951.pdf]

Journal of Health Economics and Outcomes Research

Online Supplementary Material

Retrospective Analysis of Burden of Illness of Congenital Pulmonary Valve Disease in a Large, Commercially Insured US Population. *JHEOR*. 2026;13(1):56-65. doi:10.36469/jheor.2026.155395

Table S1: Cohort Defining CPVD Diagnoses and Procedures

Table S2: Baseline Patient Characteristics by Duration of Follow-up from Birth

Table S3: CPVD-Related Inpatient Utilization by Diagnostic Group Stratification for Index Age Category Groups

Table S4: CPVD-Related Outpatient Utilization Detail

Table S5: Utilization and Costs for CPVD-related Medical Care by Duration of Follow-up from Birth

Table S6: Utilization and Costs for All-Cause Medical Care by Duration of Follow-Up from Birth

Table S7: All-Cause Annual Costs for Medical Care by Age at Index

Figure S1: Depiction of Study Design and Periods

This supplementary material has been provided by the authors to give readers additional information about their work.

This is an open-access article distributed under the terms of the Creative Commons Attribution 4.0 International License (CCBY-4.0). View this license's legal deed at http://creativecommons.org/licenses/by/4.0 and legal code at http://creativecommons.org/licenses/by/4.0/legalcode for more information.

Table S1. Cohort Defining CPVD Diagnoses and Procedures

	**Diagnosis or Procedure**
CPVD-related diagnoses	Congenital pulmonary valve insufficiency<br>Pulmonary regurgitation[1]<br>Pulmonary stenosis[1]<br>Branch pulmonary artery stenosis[1] (left pulmonary artery, right pulmonary artery)<br>Heart failure[1]<br>Right ventricular dilation/enlargement[1]<br>Right ventricular dysfunction[1]<br>Right heart failure[1]<br>Shortness of breath[2,3]<br>Right bundle branch block[1]<br>Atrial arrhythmia[1]<br>Ventricular arrhythmia[1]<br>Aortic root dilatation[4]<br>Exercise limitation/intolerance[1]<br>Liver fibrosis[5,6]<br>Tricuspid valve regurgitation[1]<br>Failure to thrive[3]<br>Growth restriction[7]
CPVD-related procedures	Primary surgical repair of anatomic defect<br>Surgical/transcatheter pulmonary valve replacement<br>Surgical implantation of right ventricle to pulmonary artery conduit<br>Surgical tricuspid valve repair<br>Surgical patch pasty of left or right pulmonary artery<br>Percutaneous balloon pulmonary valvuloplasty<br>Percutaneous branch pulmonary artery balloon angioplasty<br>Percutaneous branch pulmonary artery stent<br>Right ventricular outflow tract stent placement

Abbreviation: CPVD, congenital pulmonary valve disease.

Table S2. Baseline Patient Characteristics by Duration of Follow-up from Birth

	60-Month Follow-up	120-Month Follow-up
No. of patients, N	872	174
Length of follow-up (years), mean (SD); median [IQR]	8.1 (2.80); 7.3 [6.0-9.4]	12.7 (2.44); 11.9 [10.6-14.2]
Gender, n (%)		
Female	447 (51.3)	86 (49.4)
Male	425 (48.7)	88 (50.6)
Geographic region, n (%)		
West	127 (14.6)	24 (13.8)
South	307 (35.2)	58 (33.3)
Northeast	95 (10.9)	14 (8.0)
Midwest	242 (27.8)	63 (36.2)
Unknown	101 (11.6)	15 (8.6)
Individual-level race/ethnicity, among those with data, n (%)		
American Indian or Alaska Native[a]	<5	0 (0.0)
Asian[a]	40 (4.6)	7 (4.0)
Black or African American[a]	61 (7.0)	16 (9.2)
Hispanic or Latino of any race	63 (7.2)	13 (7.5)
Native Hawaiian or other Pacific Islander[a]	<5	0 (0.0)
White[a]	632 (72.5)	132 (75.9)
Other race[a]	21 (2.4)	<5
Unknown or undisclosed	52 (6.0)	<5
Area-level SES index[b] category, n (%)		
1	82 (9.4)	16 (9.2)
2	163 (18.7)	29 (16.7)
3	198 (22.7)	43 (24.7)
4	300 (34.4)	66 (37.9)
Missing/unknown	129 (14.8)	20 (11.5)
Selected comorbidities, n (%)		
Hypertension	89 (10.2)	17 (9.8)
Heart failure	191 (21.9)	37 (21.3)
Developmental delays	345 (39.6)	58 (33.3)
Asthma	232 (26.6)	55 (31.6)
Dyslipidemia	17 (1.9)	9 (5.2)
Ventricular arrythmia	60 (6.9)	13 (7.5)
Obesity	91 (10.4)	30 (17.2)
Pulmonary hypertension	73 (8.4)	15 (8.6)
Ischemic heart disease	42 (4.8)	11 (6.3)
Atrial fibrillation	14 (1.6)	<5
Atrial tachycardia	77 (8.8)	10 (5.7)
Chronic heart failure[c]	33 (3.8)	7 (4.0)
Cyanosis	141 (16.2)	17 (9.8)
Diabetes mellitus	19 (2.2)	7 (4.0)
Renal disease	31 (3.6)	10 (5.7)
Stroke	35 (4.0)	<5
Chronic obstructive pulmonary disease	35 (4.0)	6 (3.4)
Peripheral vascular disorders	12 (1.4)	<5
Myocardial infarction	8 (0.9)	<5
Sudden cardiac death	28 (3.2)	6 (3.4)
Liver congestion/fibrosis	6 (0.7)	<5
Right heart failure	8 (0.9)	<5
Arrhythmias (unspecified)	32 (3.7)	<5

Abbreviations: IQR, interquartile range; SDoH, social determinant of health; SES, socioeconomic status.

[a]Not Hispanic or Latino.

[b]SES index is a composite measure based on 7 SDoH variables. A score of 4 indicates top 25% of SES, and a score of 1 indicates bottom 25% of SES using all census block groups and 2017 as reference basis. Patients unable to be linked to SDoH data or with ≥1 of the 7 variables missing are categorized as unknown.

[c]Diagnosis codes are the same as used for heart failure, but chronic heart failure required ≥2 outpatient claims ≥30 days apart (no maximum).

Table S3. CPVD-Related Inpatient Utilization by Diagnostic Group Stratification for Index Age Category Groups

	By Index Age Categories (Years)						By Duration of Follow-up from Birth	
	<1 (N=5952)	1-4 (N=7111)	5-12 (N=6560)	13-21 (N=4985)	22-44 (N=5901)	45-65 (N=2298)	60 Months (N=872)	120 Months (N=174)
**Prevalence of CPVD diagnostic subgroups, n (%)**								
Double outlet right ventricle	588 (9.9)	716 (10.1)	683 (10.4)	530 (10.6)	595 (10.1)	86 (3.7)	77 (8.8)	13 (7.5)
Tetralogy of Fallot	1368 (23.0)	1764 (24.8)	2181 (33.2)	1932 (38.8)	2910 (49.3)	1127 (49.0)	199 (22.8)	34 (19.5)
Pulmonary valve atresia	512 (8.6)	623 (8.8)	690 (10.5)	559 (11.2)	547 (9.3)	104 (4.5)	89 (10.2)	20 (11.5)
Congenital pulmonary valve stenosis	3764 (63.2)	4372 (61.5)	3408 (52.0)	2266 (45.5)	1994 (33.8)	842 (36.6)	597 (68.5)	124 (71.3)
Other congenital malformations of pulmonary valve	484 (8.1)	501 (7.0)	297 (4.5)	178 (3.6)	190 (3.2)	149 (6.5)	55 (6.3)	7 (4.0)
Pulmonary infundibular stenosis	2027 (34.1)	2507 (35.3)	2094 (31.9)	1419 (28.5)	1077 (18.3)	488 (21.2)	364 (41.7)	121 (69.5)
Atresia of pulmonary artery	800 (13.4)	931 (13.1)	845 (12.9)	536 (10.8)	502 (8.5)	118 (5.1)	175 (20.1)	43 (24.7)
**CPVD-related inpatient utilization frequency**								
Patients with ≥1 CPVD-related inpatient admissions, n (%)	3169 (53.2)	1365 (19.2)	957 (14.6)	1000 (20.1)	1536 (26.0)	781 (34.0)	528 (60.6)	91 (52.3)
**Patients with ≥1 CPVD-related inpatient admission by individual CVPD diagnoses,[a] n (%)**								
Double outlet right ventricle	522 (88.8)	318 (44.4)	172 (25.2)	165 (31.1)	190 (31.9)	36 (41.9)	76 (98.7)	12 (92.3)
Tetralogy of Fallot	1202 (87.9)	444 (25.2)	446 (20.4)	477 (24.7)	792 (27.2)	435 (38.6)	194 (97.5)	34 (100.0)
Pulmonary valve atresia	463 (90.4)	307 (49.3)	231 (33.5)	193 (34.5)	191 (34.5)	27 (26.0)	87 (97.8)	19 (95.0)
Congenital pulmonary valve stenosis	1539 (40.9)	637 (14.6)	363 (10.7)	386 (17.0)	528 (26.5)	257 (30.5)	307 (51.4)	50 (40.3)
Other congenital malformations of pulmonary valve	256 (52.9)	82 (16.4)	30 (10.1)	38 (21.3)	49 (25.8)	47 (31.5)	41 (74.5)	5 (71.4)
Pulmonary infundibular stenosis	841 (41.5)	413 (16.5)	250 (11.9)	266 (18.7)	298 (27.7)	163 (33.4)	178 (48.9)	47 (38.8)
Atresia of pulmonary artery	558 (69.8)	367 (39.4)	246 (29.1)	186 (34.7)	191 (38.0)	64 (54.2)	127 (72.6)	33 (76.7)

Abbreviation: CPVD, congenital pulmonary valve disease.

[a]The percentage with CPVD-related inpatient admissions is calculated among patients with each respective CPVD diagnosis. The individual CPVD diagnoses are not mutually exclusive; it is possible to be counted in more than one diagnosis category.

Table S4. CPVD-Related Outpatient Utilization Detail

	Index Age Category (Years)					
	<1 (N=5952)	1-4 (N=7111)	5-12 (N=6560)	13-21 (N=4985)	22-44 (N=5901)	45-65 (N=2298)
**Outpatient encounters, inclusive of all[a] outpatient visits and types**						
Patients with ≥ 1 visit, N (%)	5866 (98.6)	6361 (89.5)	5855 (89.3)	4692 (94.1)	5620 (95.2)	2231 (97.1)
Outpatient services per year						
Mean (SD)	18.7 (32.1)	6.5 (20.5)	5.7 (19.2)	5.4 (19.8)	7.1 (12.2)	9.6 (15.7)
Median [IQR]	9.3 [4.0-22.5]	1.8 [0.7-4.8]	2.4 [0.9-5.2]	2.7 [1.2-5.4]	3.8 [1.8-7.7]	4.8 [2.1-10.9]
**Breakdown of outpatient visit by type**						
Office visit: Patients with ≥1 service, N (%)	5512 (92.6)	6000 (84.4)	5563 (84.8)	4470 (89.7)	5296 (89.7)	2118 (92.2)
Office visits per year						
Mean (SD)	8.1 (13.2)	1.8 (2.8)	2.0 (5.3)	2.1 (7.3)	2.7 (4.2)	3.6 (6.0)
Median [IQR]	4.9 [2.3-10.1]	0.9 [0.3-2.0]	1.2 [0.5-2.2]	1.2 [0.6-2.2]	1.6 [0.7-3.0]	2.1 [0.9-4.1]
Imaging: Patients with ≥1 service, N (%)	5186 (87.1)	5408 (76.1)	5176 (78.9)	4214 (84.5)	4902 (83.1)	1825 (79.4)
Imaging encounters per year						
Mean (SD)	6.4 (15.3)	1.3 (2.4)	2.0 (10.5)	2.2 (10.0)	2.5 (4.9)	2.0 (4.1)
Median [IQR]	4.0 [2.0-7.8]	0.7 [0.3-1.5]	1.0 [0.3-2.1]	1.1 [0.4-2.2]	1.3 [0.5-2.7]	1.1 [0.3-2.3]
Medication related visit: patients with ≥1 visit, N (%)	2232 (37.5)	2526 (35.5)	1770 (27.0)	1420 (28.5)	1578 (26.7)	996 (43.3)
Lab test service: Patients with ≥1 service, N (%)	1537 (25.8)	1847 (26.0)	1821 (27.8)	1894 (38.0)	2850 (48.3)	1543 (67.1)
Physician other service: patients with ≥1 visit, N (%)	1369 (23.0)	1628 (22.9)	1237 (18.9)	982 (19.7)	1290 (21.9)	773 (33.6)
Procedures: Patients with ≥1 visit, N (%)	1152 (19.4)	1708 (24.0)	1566 (23.9)	1053 (21.1)	1131 (19.2)	737 (32.1)

Abbreviations: CPVD, congenital pulmonary valve disease; IQR, interquartile range.
[a]Outpatient encounters include office visits, procedures, tests (lab, imaging, medication and related services, durable medical equipment, physical therapy/occupational therapy/speech therapy, physician–other, tests–other), and other. Categories with the highest frequencies are displayed in the table.

Table S5. Utilization and Costs for CPVD-related Medical Care by Duration of Follow-up from Birth

**Costs/Utilization**		**60 Months (N=872)**	**120 Months (N=174)**
**Inpatient**			
Patients with ≥1 inpatient admission, N (%)		528 (60.6)	91 (52.3)
Admissions/year[a]	Mean (SD)	0.3 (0.51)	0.2 (0.30)
	Median [IQR]	0.2 [0.0-0.4]	0.1 [0.0-0.2]
Admission days per year, cumulative[b]	Mean (SD)	9.3 (13.60)	4.7 (7.77)
	Median [IQR]	4.0 [1.6-11.0]	1.8 [0.7-4.9]
Total cost[a] per year, $	Mean (SD)	52,993 (104,694)	25,077 (63,096)
	Median [IQR]	9780 [0-55,945]	313 [0-19,019]
**Emergency department**			
Patients with ≥1 emergency department visit, N (%)		182 (20.9)	34 (19.5)
Total cost[a] per year, $	Mean (SD)	181 (563)	91 (320)
	Median [IQR]	0 [0-0]	0 [0-0]
**Outpatient[c]**			
Patients with ≥1 outpatient service, N (%)		870 (99.8)	173 (99.4)
Total cost[a] per year, $	Mean (SD)	6028 (11,917)	3301 (7923)
	Median [IQR]	1742 [626-6482]	711 [302-2663]
**Pharmacy**			
Patients with ≥1 pharmacy prescription fill, N (%)		64 (7.3)	14 (8.0)
Total cost[a] per year, $	Mean (SD)	334 (2670)	423 (4264)
	Median [IQR]	0 [0-0]	0 [0-0]
**Total costs[a]**			
Total cost[a] per year, $	Mean (SD)	59,537 (111,982)	28,893 (69,019)
	Median [IQR]	13,690 [918-66,625]	2852 [400-23,858]
Years in follow-up period		5	10
Total mean cost[a] for follow-up period,[d] $		297,685	288,930

[a]Calculated for the total number of patients in the respective subgroups.
[b]Calculated among patients with ≥1 inpatient admission.
[c]Outpatient includes office visits, procedures, texts, medication-related services, durable medical equipment, and various therapy services. All costs are adjusted to 2023 United States dollars provided by the Bureau of Labor Statistics.
[d]Total mean cost for follow-up period = (Years in follow-up cohort) × (Total mean cost per year).
Abbreviations: CPVD, congenital pulmonary valve disease; IQR, interquartile range; SD, standard deviation.

Table S6. Utilization and Costs for All-Cause Medical Care by Duration of Follow-Up from Birth

**Costs/Utilization**		**60 Months (N=872)**	**120 Months (N=174)**
**Inpatient**			
Patients with ≥1 inpatient admission, N (%)		563 (64.6)	109 (62.6)
Admissions/year[a]	Mean (SD)	0.4 (0.64)	0.2 (0.39)
	Median [IQR]	0.2 [0.0-0.6]	0.1 [0.0-0.3]
Admission days per year, cumulative[b]	Mean (SD)	9.2 (13.83)	4.4 (7.72)
	Median [IQR]	3.6 [1.6-11.0]	1.5 [0.6-4.4]
Total cost[a] per year, $	Mean (SD)	54,966 (107,665)	26,545 (63,863)
	Median [IQR]	11,266 [0-58,535]	2067 [0-19,785]
**Emergency department**			
Patients with ≥1 emergency department visit, N (%)		574 (65.8)	133 (76.4)
Total cost[a] per year, $	Mean (SD)	742 (1443)	538 (957)
	Median [IQR]	265 [0-833]	210 [49-551]
**Outpatient[c]**			
Patients with ≥1 outpatient service, N (%)		872 (100.0)	174 (100.0)
Total cost[a] per year, $	Mean (SD)	15,484 (26,256)	12,179 (25,805)
	Median [IQR]	5871 [2835-15,986]	3397 [1684-10,492]
**Pharmacy**			
Patients with ≥1 pharmacy prescription fill, N (%)		859 (98.5)	174 (100.0)
Total cost[a] per year, $	Mean (SD)	1640 (5212)	2127 (8625)
	Median [IQR]	157 [53-943]	150 [64-814]
**Total costs[a]**			
Total cost[a] per year, $	Mean (SD)	72,846 (128,710)	41,390 (86,262)
	Median [IQR]	20,085 (4254-81,413)	8185 (2402-36,376)
Years in follow-up period		5	10
Total mean cost[a] for follow-up period,[d] $		364,230	413,900

[a]Calculated for the total number of patients in the respective subgroups.
[b]Calculated among patients with ≥1 inpatient admission.
[c]Outpatient includes office visits, procedures, texts, medication-related services, durable medical equipment, and various therapy services. All costs are adjusted to 2023 US dollars provided by the Bureau of Labor Statistics.
[d]Total mean cost for follow-up period = (Years in follow-up cohort) × (Total mean cost per year).
Abbreviations: CPVD, congenital pulmonary valve disease; IQR, interquartile range; SD, standard deviation.

Table S7. All-Cause Annual Costs for Medical Care by Age at Index

	**Index Age Category (Years)**					
	**<1 (N=5952)**	**1-4 (N=7111)**	**5-12 (N=6560)**	**13-21 (N=4985)**	**22-44 (N=5901)**	**45-65 (N=2298)**
**All-cause inpatient costs[a,b] total $ per year**						
Mean (SD)	271,118 (738,978)	21,415 (176,936)	14,835 (128,914)	22,016 (366,217)	21,631 (116,897)	27,158 (120,837)
Median [IQR]	10,800 [0-242,932]	0 [0-0]	0 [0-0]	0 [0-0]	0 [0-8,458]	0 [0-12,054]
**All-cause emergency department costs[a,b] total $ per year**						
Mean (SD)	1,156 (5,669)	712 (3,087)	561 (2,606)	899 (4,877)	1,155 (5,372)	1,127 (4,653)
Median [IQR]	0 [0-0]	0 [0-568]	0 [0-337]	0 [0-547]	0 [0-643]	0 [0-736]
**All-cause outpatient[c] costs[a,b] total $ per year**						
Mean (SD)	28,748 (50,439)	12,858 (29,868)	11,492 (30,835)	11,210 (31,655)	13,647 (39,802)	16,925 (34,590)
Median [IQR]	13,982 [6,810-33,561]	3,840 [1,722-11,299]	3,803 [1,603-9,541]	4,465 [1,958-10,158]	5,916 [2,645-12,972]	7,615 [3,480-16,541]
**All-cause skilled nursing facility costs[a,b] total $ per year**						
Mean (SD)	10 (775)	20 (1,575)	0 (0)	2 (158)	1 (60)	90 (1,971)
Median [IQR]	0 [0-0]	0 [0-0]	0 [0-0]	0 [0-0]	0 [0-0]	0 [0-0]
**All-cause total pharmacy costs[a,b] total $ per year**						
Mean (SD)	2,011 (8,123)	1,318 (6,079)	2,114 (16,413)	2,593 (16,872)	2,384 (11,566)	5,754 (16,860)
Median [IQR]	95 [0-600]	85 [15-358]	102 [17-520]	172 [25-768]	280 [51-1,172]	1,278 [336-4,724]
**All-cause total costs[a,b] $ per year**						
Mean (SD)	303,043 (747,650)	36,323 (186,666)	29,002 (138,781)	36,720 (373,254)	38,818 (141,033)	51,055 (135,002)
Median [IQR]	53,743 [9,619-289,930]	5,111 [2,155-19,793]	5,028 [1,995-16,538]	6,835 [2,647-22,011]	11,004 [4,210-30,752]	15,831 [6,103-45,268]
Years in cohort	1	4	8	9	23	21
Total for period	$303,043	$145,292	$232,016	$330,480	$892,814	$1,072,155
**Projected Lifetime all-cause healthcare costs through age 65: $2,975,800**						

[a]All costs are adjusted to 2023 United States dollars as per the most recent medical care price index information provided by the Bureau of Labor Statistics.
[b]The average costs are calculated for the total number of members in the respective cohorts.
[c]Outpatient costs include office visits, procedures, tests (lab, imaging, medication related services, durable medical equipment, physical/occupational/speech therapy).
Abbreviations: IQR, interquartile range; SD, standard deviation.

Supplemental Figure S1. Depiction of Study Design and Periods

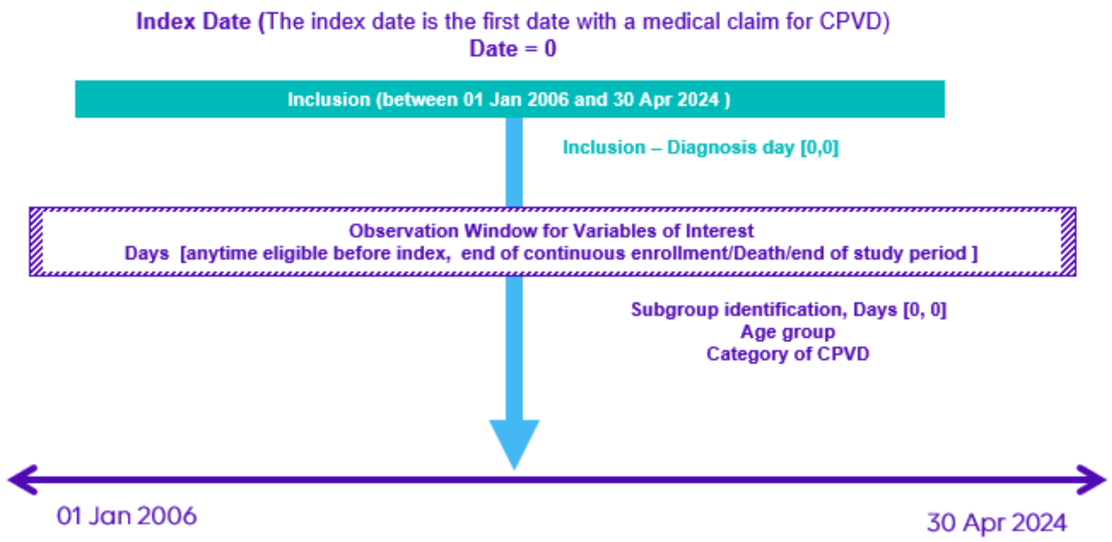

Abbreviation: CPVD, congenital pulmonary valve disease.

REFERENCES

1. Geva T, Wald RM, Bucholz E, et al. Long-Term Management of Right Ventricular Outflow Tract Dysfunction in Repaired Tetralogy of Fallot: A Scientific Statement From the American Heart Association. *Circulation.* 2024;150(25):e689-e707. 10.1161/cir.0000000000001291

2. Chaturvedi RR, Redington AN. Pulmonary regurgitation in congenital heart disease. *Heart.* 2007;93(7):880-889. 10.1136/hrt.2005.075234

3. Shekhar S, Agrawal A, Pampori A, Lak H, Windsor J, Ramakrishna H. Mortality in Adult Congenital Heart Disease: Analysis of Outcomes and Risk Stratification. *J Cardiothorac Vasc Anesth.* 2022;36(8 Pt B):3379-3388. 10.1053/j.jvca.2022.03.010

4. Cuypers JAAE, Menting ME, Konings EEM, et al. Unnatural History of Tetralogy of Fallot. *Circulation.* 2014;130(22):1944-1953. doi:10.1161/CIRCULATIONAHA.114.009454

5. Mordaka M, Ciupińska J, Jabłkowska A, et al. Non-invasive assessment of hepatic steatosis and fibrosis and echocardiographic parameters of the function of the right ventricle. *Folia Med Cracov.* 2025;65(2):93-99. 10.24425/fmc.2025.156127

6. Ravndal MEH, Borgwardt L, Juul K, et al. Liver fibrosis in patients with tetralogy of Fallot, an unrecognised complication? *Cardiol Young.* 2021;31(11):1796-1806. 10.1017/s1047951121000901

7. Dragomir I, Vasilescu DI, Dan AM, et al. Tetralogy of Fallot: The Burden of Pulmonary Atresia in the NICU Set-Up: Two Case Reports and a Literature Review. *Children (Basel).* 2025;12(6). 10.3390/children12060780
